# Prognostic Role of Demographic, Injury and Claim Factors in Disabling Pain and Mental Health Conditions 12 Months after Compensable Injury

**DOI:** 10.3390/ijerph17197320

**Published:** 2020-10-07

**Authors:** Thi L. Nguyen, Katharine S. Baker, Liane Ioannou, Behrooz Hassani-Mahmooei, Stephen J. Gibson, Alex Collie, Jennie Ponsford, Peter A. Cameron, Belinda J. Gabbe, Melita J. Giummarra

**Affiliations:** 1 Department of Epidemiology and Preventive Medicine, School of Public Health and Preventive Medicine, Monash University, Wellington Rd, Clayton, VIC 3800, Australia; Thi.Nguyen7@monash.edu (T.L.N.); liane.ioannou@monash.edu (L.I.); behrooz.hassani.mahmooei@monash.edu (B.H.-M.); alex.collie@monash.edu (A.C.); peter.cameron@monash.edu (P.A.C.); belinda.gabbe@monash.edu (B.J.G.); 2 School of Psychological Sciences and Monash Institute of Cognitive and Clinical Neurosciences, Monash University, Faculty of Medicine, Nursing and Health Sciences, 27 Rainforest Walk, Clayton, VIC 3800, Australia; katharine.baker@uqconnect.edu.au (K.S.B.); jennie.ponsford@monash.edu (J.P.); 3 Caulfield Pain Management and Research Centre, Caulfield Hospital, 260–294 Kooyong Rd, Caulfield, VIC 3162, Australia; profstephengibson@outlook.com; 4 Monash-Epworth Rehabilitation Research Centre, Epworth Hospital, Richmond, 89 Bridge Rd, Richmond, VIC 3121, Australia; 5 Health Data Research UK, Swansea University Medical School, Swansea University, Wales, Sketty, Swansea SA2 8QA, UK

**Keywords:** compensation, insurance, traumatic injury, disability, injury, pain, mental health

## Abstract

Identifying who might develop disabling pain or poor mental health after injury is a high priority so that healthcare providers can provide targeted preventive interventions. This retrospective cohort study aimed to identify predictors of disabling pain or probable mental health conditions at 12 months post-injury. Participants were recruited 12-months after admission to a major trauma service for a compensable transport or workplace injury (*n* = 157). Injury, compensation claim, health services and medication information were obtained from the Victorian Orthopaedic Trauma Outcome Registry, Victorian State Trauma Registry and Compensation Research Database. Participants completed questionnaires about pain, and mental health (anxiety, depression, posttraumatic stress disorder) at 12 months post-injury. One third had disabling pain, one third had at least one probable mental health condition and more than one in five had both disabling pain and a mental health condition at 12 months post-injury. Multivariable logistic regression found mental health treatment 3–6 months post-injury, persistent work disability and opioid use at 6–12 months predicted disabling pain at 12 months post-injury. The presence of opioid use at 3–6 months, work disability and psychotropic medications at 6–12 months predicted a mental health condition at 12 months post-injury. These factors could be used to identify at risk of developing disabling pain who could benefit from timely interventions to better manage both pain and mental health post-injury. Implications for healthcare and compensation system are discussed.

## 1. Introduction

Seventy-five percent of injured people report chronic pain up to 3 years post-injury [1,2]. Chronic pain has been associated with having more mental health symptoms [3,4], work disability [5,6], increased health care utilisation [7], higher compensation claim cost and longer claim duration [6,8]. Longitudinal studies demonstrate that approximately one in five injured people have a poor recovery trajectory across psychological, pain and functional outcomes over the first 12–24 months after whiplash injury [9,10]. While a similar proportion of people report disabling pain (i.e., pain that is intense, frequent and activity limiting) at 12 months [1] and 3 years after serious injury [2], up to 65% of people report persistent or worsening problems with pain or mental health over the first 2 years after serious road traffic injury [11]. Several risk factors for chronic pain after injury are already known, including pre-injury factors, such as middle–older age [6,12], female sex [6,13], lower education [14,15,16], comorbidities [4,14,16,17], preinjury mental health [11], injury severity, type and body region (e.g., lower limb, back and trunk) [1]. Psychosocial factors such as perceived fault [18], blame and injustice [19] also play a role in injury recovery. Moreover, psychological distress is common after road traffic injury, particularly after whiplash and spinal cord injury [20]. While mental health conditions frequently co-occur with chronic pain, their occurrence does not seem to be specifically related to injury severity [13,21]. Rather acute reactions to the trauma [21,22,23] and fault attributions [18] appear to play a pertinent role in prolonged distress after injury. 

Regardless of the type of injury sustained, it is frequently found that pain and mental health outcomes are worse in people who claim or pursue compensation [24,25,26]. A number of factors seem to be involved in the so-called “compensation health effect”. While malingering or symptom exaggeration may be present in some claimants [27], it seems that adverse outcomes from compensable injury, and aspects of compensation-related processes, may exacerbate the impact of the injury on both pain and mental health outcomes [28]. In particular, disability, pain and mental health after compensable injury are associated with subjective experience of claim-related stress, particularly due to claim delays [29,30], undergoing independent medical examinations to determine the severity of functional impairment [31], as well as attributions of fault or consulting a lawyer [26]. Moreover, perceptions of injustice [32], having a sense of entitlement or embodiment of a “victim” role [33] may play a role. Many of these associations are likely to be bidirectional given that processes like independent medical examinations are often requested because a person reports persistent pain and/or psychological injury in order to determine the veracity of that self-report. Moreover, many processes occur late in the claim period or only in fault-based schemes. While they may not be causal, these characteristics may nonetheless help to proactively identify people who are at greater risk of having a poor recovery.

The primary aim of this retrospective cohort study was to investigate whether information available to compensation schemes within the first 12 months post-injury, in addition to demographic and injury characteristics known at the time of injury, could enable better identification of who is at greater risk of reporting chronic disabling pain or clinically elevated symptoms of depression, anxiety or posttraumatic stress disorder (PTSD), at 12 months after injury. The broader purpose of the study was to inform the development and implementation of screening procedures within compensation schemes to identify people at risk of disabling pain so that they could receive timely interventions to augment their recovery.

## 2. Study Setting and Context

This study was conducted in Victoria, Australia, and the results should be considered in light of the design of the Victorian transport injury compensation scheme (Transport Accident Commission, TAC) and workers’ compensation scheme (WorkSafe Victoria, WSV). The TAC and WSV differ from compensation schemes in many other states of Australia and other countries, particularly for transport-related schemes where eligibility to claim may be conditional upon identifying another person at fault. 

The TAC is a statutory compensation scheme for supporting people who have sustained injuries that involve a motorised vehicle, train or tram. An injured person is eligible to a claim the costs of their medical and rehabilitation treatment irrespective of who is at fault. The TAC covers the cost of healthcare provided by hospital and medical services, allied health practitioners, rehabilitation services, attendant care, home support and medical aids, to name a few. Claimants can also receive full or partial loss of earnings support commencing five or more days after the injury if they were aged 15 years or older at the time of injury, their injuries prevent them from returning to work and they have a certificate of capacity indicating that they are unable to return to work in full capacity. For continued loss of earnings support, and other healthcare benefits (e.g., ongoing therapeutic/rehabilitation services, surgeries past 3 months post-injury), claimants may need to attend an independent medical examination (IME) with a TAC-nominated medical specialist to determine whether the injury is still a cause of the client’s complaints and whether the proposed treatment is clinically justified. An IME may also be required to assist the TAC to determine the client’s impairment level, once their injury has stabilised, in accordance with the American Medical Association Guides for the provision of Impairment Assessment [34]. Permanent impairment is classified as a demonstrable impairment of functional capacity greater than 11%, and includes both physical and psychological conditions. The IME findings assist the TAC to determine the level of impairment benefit, and for persons whose injury is determined at 50% whole person impairment, ongoing treatment, loss of earnings, or loss of earning capacity (LOEC) benefits. For people who hold private health insurance and have made a claim on their insurance for a transport injury-related treatment, the private health insurance company can request reimbursement of expenses from the TAC at TAC rates. Depending on the private health insurance coverage of each person, this dictates the type of services covered.

People will have reduced entitlement to loss of earnings from the TAC if they were committing an offence (e.g., drink driving offences or culpable/dangerous driving causing death) at the time of injury. Moreover, the TAC will not pay for the treatment of conditions unrelated to the transport injury, though it will pay for any aggravation caused by the injury to any pre-existing conditions or injuries. Finally the TAC will not accept liability for the claim if the person is entitled to compensation through another statutory insurance scheme.

WorkSafe Victoria (WSV) is a no-fault compensation scheme that manages claims for health and income support by workers injured during the course of their employment. When a worker sustains an injury in a road or rail-related incident during the course of their employment, the worker receives compensation benefits from WSV, but could also be entitled to common law damages through WorkSafe, TAC or both schemes, depending on the circumstances of the injury. Eligibility for the types of benefits payable from the TAC and WSV differ. People with a compensation claim may receive some treatments and medications through the publicly funded Medicare Benefits Schedule or Pharmaceutical Benefits Scheme [35,36], respectively.

## 3. Materials and Methods

### 3.1. Patient Recruitment

Potential participants were eligible if they were admitted to The Alfred, one of two adult major trauma services in Victoria, Australia, and if they were registered to the Victorian Orthopaedic Trauma Outcomes Registry (VOTOR) [37] or the Victorian State Trauma Registry (VSTR) [38]. Patients were invited to participate during the registry interview at 12 months post-injury. People who consented to participate were asked to complete additional outcome measures about their pain, mental health and compensation experiences. Participants were only included in the present study if the hospital admission was funded by the TAC or WorkSafe Victoria, or if people reported lodging a claim in our extended 12-month follow-up interview. All claims were then confirmed with the TAC or WorkSafe Victoria.

**Figure 1:**
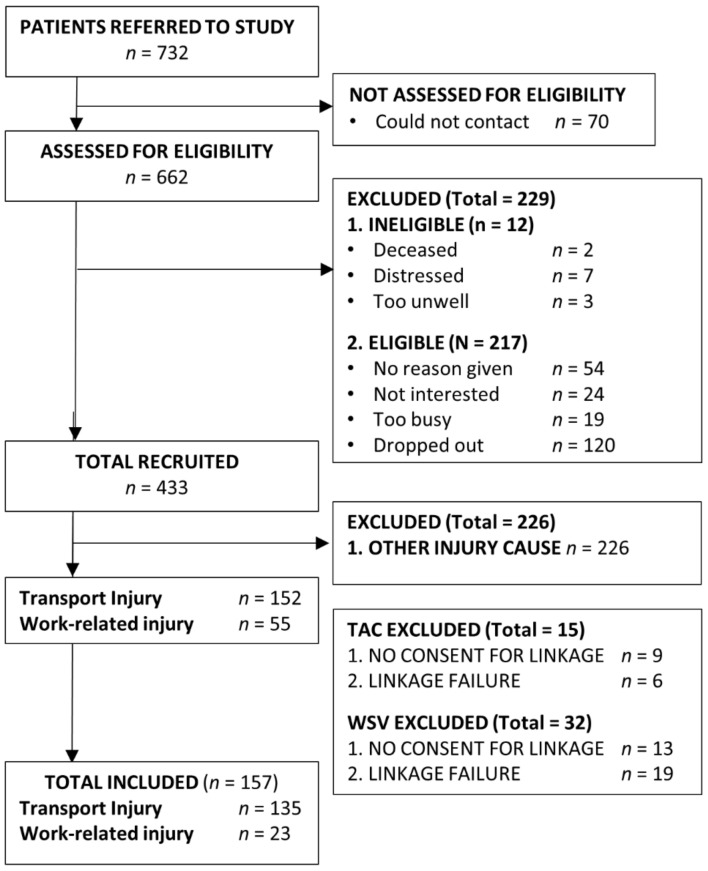
Participant recruitment flow chart. Note: One participant had both Transport Accident Commission (TAC) and WorkSafe Victoria (WSV) claims data.

The VSTR includes data on all trauma admissions that meet major trauma criteria, defined as traumatic injury resulting in (a) death from injury; (b) admission to intensive care unit for ≥ 24 h and being mechanistically ventilated; (c) an injury severity score (ISS) greater than 12; or (d) urgent surgery for intracranial, intrathoracic or intra-abdominal injury, or fixation of pelvic or spinal fractures. Patients are included in VOTOR if they sustained orthopaedic (bone or soft tissue) injuries resulting in admission to hospital for >24 h. Patients with soft tissue injuries that were managed conservatively do not enter VOTOR and therefore were not eligible for participation in the present study. Patients who were distressed and had difficulty completing the registry interviews or who required a proxy to complete their registry interview (e.g., due to cognitive impairment from brain injury) were not invited to participate. A recruitment flowchart is presented in Figure 1.

### 3.2. Data Linkages

Participants consented to the linkage of information collected from questionnaires administered in this study with information about their injury, compensation claim and 12-month outcomes data from VSTR, VOTOR and the Compensation Research Database (CRD). The source of each variable is specified in Table S1. The dataset for this project is not available for public release, in accordance with the data access agreements with the VSTR, VOTOR, TAC and WSV. The VSTR and VOTOR contain information about patient demographics, admission, trauma and surgical procedures at baseline from hospital medical records, and outcomes that are assessed at 6, 12 and 24 months post-injury through structured interviews. For this study, data extracted from the VSTR and VOTOR included patient demographics (age, sex, level of education and work type before injury), injury characteristics (injury date, severity and body region using the abbreviated injury severity (AIS) scores, injury severity score (ISS), and injury classifications based on the International Statistical Classification of Diseases and Related Health Problems, Tenth Revision, Australian Modification (ICD-10-AM)), hospital admission details (i.e., length of hospital stay, intensive care unit admission and discharge destination) and pre-injury health (EQ-5D VAS, comorbidities). To characterise injury severity, we used the sum of AIS scores consistent with a previous study [1,2] given that the ISS shows a poor association with pain and mental health symptoms 12 months after injury [1,39]. Pre-injury comorbidities were based on ICD-10-AM diagnoses at the time of injury, including pre-injury mental health conditions and substance use conditions using the Chapter V “F codes” [40]. Participants also self-reported their comorbid conditions at 12 months post-injury as part of the study questionnaire.

The CRD comprised all claims data (e.g., benefit types, dates of service/payment, claim classifications) from transport and workplace injuries that resulted in a compensation claim to the TAC or WSV [41] and are recorded prospectively in real-time when the respective scheme has made a payment. Deterministic linkage using a claim number was used to obtain TAC claims data. The WSV claims data were identified by WSV using participant name, sex, date of birth and date of injury. One TAC client had no benefits recorded and was excluded, and claims records for 25 patients who reported that they sustained a road traffic injury or workplace injury could not be identified (see Figure 1). 

The claims data used for this study included benefits received for income replacement, lump sum payments for permanent impairment, prescription medication costs, independent medical examinations (IMEs) and health care provided by hospital and medical services, allied health practitioners and rehabilitation services. Service items were only included if they involved face-to-face clinical interaction. Items relating to clinical report writing, travel, childcare or domestic services, freight and administrative costs were excluded, consistent with previous research [42]. To provide context for the types of healthcare services received by the current cohort, Table 1 summarises the total number and cost of therapeutic medical, paramedical and pharmaceutical items. Flags were generated to indicate whether specific types of benefits or treatments were received, and the total cost of services was calculated rather than the number of treatments received given that more invasive or intensive treatments (e.g., surgery) are not likely to be equivalent in their association with pain and mental health outcomes to a less costly health service (e.g., a psychology or physiotherapy session). 

### 3.3. Materials and Procedure

The study protocol was approved by the Alfred Hospital (study: 290/13) and Monash University (study: CF13/3276—2013001633) Human Research Ethics Committees, and all participants gave written informed consent. The pain and psychological outcome measures were administered by study researchers at 12–14 months after injury through telephone interview, online, or in hard-copy, according to participant preference. 

#### 3.3.1. Demographics

Area level socioeconomic status was measured with the Index of Relative Socioeconomic Disadvantage (IRSD) decile [44]. Low IRSD scores indicate relatively greater neighbourhood disadvantage (i.e., many households with low income, low level of qualifications, low skill occupations), and high scores reflect lower levels of neighbourhood disadvantage (i.e., few households with low income, few people with no qualifications or employed in low skilled occupations). Remoteness was classified according to the Accessibility/Remoteness Index of Australia (ARIA) [45]. As only a small number of participants lived in outer regional/remote areas, participants were simply classified as residing in major cities or in regional areas.

#### 3.3.2. Pain and Pain-Related Disability

The Brief Pain Inventory (BPI) [46] was used to assess pain intensity and pain interference. The Roland-Morris Disability Questionnaire (RMDQ) [47] was used to measure physical disability due to pain. In this study, the criteria for “chronic and disabling pain” were defined as a BPI severity score of ≥ 4 and moderate–severe pain-related disability (i.e., pain interference ≥ 4 or RMDQ ≥ 7) given that a threshold of ≥ 4/10 is associated with greater analgesic requirements and subjective classification of moderate–severe pain [48,49].

#### 3.3.3. Mental Health

Symptoms of mental health conditions were measured with the Hospital Anxiety and Depression Scale (HADS) [50] and the Posttraumatic Stress Disorder Checklist (PCL-C) [51]. Participants were classified as having a mental health condition if they had symptoms indicative of moderate–severe anxiety or depression (i.e., ≥ 11) or probable PTSD (i.e., total scores > 35 [52] and satisfying all five DSM-5 criteria [53].

#### 3.3.4. Compensation Scheme Experience

In order to examine the potential association between compensation scheme experience and pain or mental health outcomes, participants rated 14 statements about their experience of compensation-related procedures, interactions and decisions from 1 = “strongly disagree” to 5 = “strongly agree”. These statements have been described in detail elsewhere [54]. An average score was calculated across items in each of the three subscales that measured negative procedural experiences, perceiving that compensation supported recovery, or having positive procedural experiences. Subscale scores of >3/5 were considered to be indicative of agreeing or strongly agreeing with the items in the respective subscale. Participants also reported whether they consulted a lawyer within the first 12 months post-injury, and whether they were at fault for their injury.

### 3.4. Data Analysis

The data were analysed in IBM SPSS Statistics, Version 23.0 (New York, United States). Less than 1% of questionnaire item responses were missing. The mental health questionnaires were missing for one participant. For the remaining participants, if a single questionnaire item was missing, the unweighted mean of the remaining items was imputed to enable accurate calculation of subscale scores. Participants missing more than one item on a subscale were coded as missing for that measure consistent with the scale scoring recommendations and methods used in previous studies [55].

Descriptive and univariate statistics (logistic regression) were used to examine which cohort characteristics increased the odds of reporting chronic and disabling pain or a mental health condition 12 months after injury, reported as the odds ratio (OR) and corresponding 95% confidence interval (95% CI). In addition to baseline characteristics, we examined whether disabling pain and mental health outcomes were associated with work disability (i.e., receiving one or more loss of earnings payment), total healthcare use (i.e., cost of all health service episodes, log transformed due to non-normal distribution) and opioid medication for pain or psychotropic medication for mental health (i.e., one or more script) in four key time periods: week 1, < 3 months but excluding week 1, 3–6 months and 6–12 months. Any characteristics that were significant at *p* ≤ 0.10 were included in the final multivariable analysis until the assumptions were met for lack of multicollinearity, sufficient case to variables (defined below), likely “causal” pathways (e.g., IMEs were considered most likely to arise because of pain/mental health) and confounding relationships (e.g., engaging a lawyer was most likely an outcome of pain or mental health, and was expected to be confounded by fault and impairment). For simplicity and due to relatively small numbers of cases receiving an impairment payment in the first 12 months post-injury, we examined “impairment” as a baseline characteristic. We acknowledge, however, that impairment level can be determined at any time following injury and can reflect the level of disability or incapacity due to the injuries sustained as well as the persistence of problems like pain or mental health conditions. 

Multivariable logistic regression analyses examined which prognostic factors during the first 12 months after injury increase the odds of reporting chronic disabling pain or a mental health condition 12–14 months after injury. The amount of variance that the predictors explained in the outcomes was calculated using Nagelkerke’s adjusted *R^2^*. The specificity and sensitivity of predicted group membership and the area under the curve (AUC) were calculated to determine how well the models predicted group membership. Given that 33.8% of participants had disabling pain and 38.5% had symptoms of at least one mental health condition, the acceptable number of predictors was five for the chronic pain outcomes analysis and seven for the mental health outcomes model based on the recommendations from Peduzzi, et al. [56] for multivariable logistic regression where *N* = 10 * number of predictors/proportion with the identified outcome. We chose to include six predictors in the chronic pain model, one more than recommended, in order to adequately control for injury severity, which seemed to be robust given that the diagnostic statistics for both analyses showed that all cases had a Cook’s Distance of <1, and fewer than 5% of the normalised residuals and deviance statistics fell outside ±1.96 [57].

## 4. Results

### 4.1. Cohort Overview

Out of 433 participants in the larger study, 157 had accepted compensation claims and consented to linkage with their claims data (Figure 1). Thirty-one people participated through telephone interview, 69 participated online, and 57 completed hard-copy questionnaires. Participants who were included were predominantly male (*n* = 118, 75.2%) and aged 18–67 years (*M* = 42.99, *SD* = 14.44). The participant characteristics are summarised in Table 2, and the prevalence of pain and mental health outcomes at 12 months are detailed in Table 3. Most participants had sustained a fracture (*n* = 149, 94.9%), with injuries most commonly affecting lower extremities, the thorax, upper extremities and the spine (Table 3). The average ISS was 17.79 (*SD* = 11.69, range: 2–59), the AIS severity score (sum) was 13.35 (*SD* = 10.63) on average and participants had injuries to a median of three body regions (IQR = 2). Most participants had sustained their injury after a motor vehicle crash (*n* = 135, TAC claimants) and 23 had workplace injuries (WSV claimants). Two-thirds (*n* = 103, 65.6%) of participants reported that they were not at fault for their injury. 

### 4.2. Predictors of Disabling Pain or Mental Health Conditions

Univariate logistic regressions were used to examine which demographic, injury and claim factors were associated with disabling pain, or symptoms of a mental health condition (refer to Tables S2 and S3 for all results). The odds of have a mental health condition were four times higher in participants who had only completed secondary school education (95%CI: 1.37, 11.92) relative to university education. Participants who did not return to work by 12 months post-injury had 6.3-fold higher odds of disabling pain (95%CI: 2.89, 13.34) and 2.9-fold higher odds of a mental health condition (95%CI: 1.43, 6.06) at 12 months compared to people who returned to work. However, the 95% CIs were very wide suggesting variability in these associations.

Thirty five percent of participants had one or more comorbid conditions. Only 10.2% of people had a prior mental health condition and 5.7% had a prior substance use condition. Comorbid conditions did not increase the odds of having disabling chronic pain (OR = 1.73, 95%CI: 0.87, 3.42) or a mental health condition 12 months post-injury (OR = 0.77, 95%CI: 0.39, 1.53). Likewise, having a prior mental health condition or substance use condition were not associated with having disabling chronic pain (prior mental health: OR = 0.63, 95%CI: 0.19, 2.04; prior substance use: OR = 0.54, 95%CI: 0.11, 2.71) or a mental health condition 12 months post-injury (prior mental health: OR = 1.28, 95%CI: 0.45, 3.63; prior substance use: OR = 3.44, 95%CI: 0.83, 14.33).

Injury severity was associated with higher odds of disabling pain, including the sum of AIS coded injuries (OR = 1.03, 95%CI: 1.00, 1.07), the number of body regions with one or more moderate to severe injuries (OR = 1.37, 95%CI: 1.06, 1.76), having a hospital length of stay of two or more weeks compared with 1-2 days (OR = 4.93, 95%CI: 1.58, 15.38) and if the claimant was discharged to rehabilitation compared with home (OR = 2.25, 95%CI: 1.15, 4.42). Indicators of injury severity did not increase the odds of having a mental health condition.

Forty-four (28.0%) participants received an impairment lump sum payment within 12 months of injury. Participants who received an impairment payment had 6.2-fold higher odds of having disabling pain (95%CI: 2.89, 13.15) and 2.9-fold higher odds of a mental health condition (95%CI: 1.41, 5.94) compared to participants without an impairment payment. Thirty-three (21.0%) participants had an IME in the first 12 months, which occurred between 34 to 356 days post-injury (*M* = 243 days, *SD* = 87 days). People who had an IME during the first 12 months post-injury had 3.0-fold higher odds of having disabling pain (95%CI: 1.39, 6.72) and 4.5-fold higher odds of a mental health condition (95%CI: 1.97, 10.14) at 12 months post-injury. People who engaged a lawyer within 12 months post-injury had 4.8-fold higher odds of disabling pain (95%CI: 2.34, 9.84) and 2.86-fold higher odds of a mental health condition (95%CI: 1.44, 5.67) at 12 months post-injury. 

The majority of participants indicated that compensation had supported their recovery (*n* = 125, 85.6%, missing *n* = 11) and that they had experienced positive aspects of compensation procedures (*n* = 114, 77.0%, missing *n* = 9); however, 69 (46.0%, missing *n* = 7) people endorsed the statements relating to negative procedural experiences. Every one point increase in perceptions that compensation supported recovery was associated with 47 percent lower odds of having chronic disabling pain (95%CI: 0.35, 0.80) and 55 percent lower odds of a mental health condition (95%CI: 0.30, 0.70), and every one point increase in positive procedural experiences was associated with 57 percent reduced odds of having a mental health condition (95%CI: 0.28, 0.67). Every one point increase in the perception of having “negative procedural experiences” during the first 12 months was associated with 78% higher odds of chronic disabling pain (*p* < 0.001, 95%CI: 1.29, 2.45), and 2.1-fold higher odds of a mental health condition (95%CI: 1.49, 2.91) at 12 months. Specific compensation-related ratings at 12 months post-injury showed that people had higher odds of having a chronic pain condition if they found the compensation claims process stressful (OR = 4.54, 95%CI: 2.10, 10.31) and felt that they had to keep proving their disability (OR = 3.00, 95%CI: 1.43, 6.28), but being unhappy with their compensation claim did not significantly increase the odds of having a chronic pain condition (OR = 1.73, 95%CI: 0.66, 4.55). Likewise, people had higher odds of having a mental health condition if they found the compensation claims process to be stressful (OR = 6.33, 95%CI: 2.76, 14.56), felt that they had to keep proving their disability (OR = 4.09, 95%CI: 1.94, 8.66), or if they were not happy with their compensation claim (OR = 2.58, 95%CI: 1.00, 6.61; Table S2).

**Figure 2:**
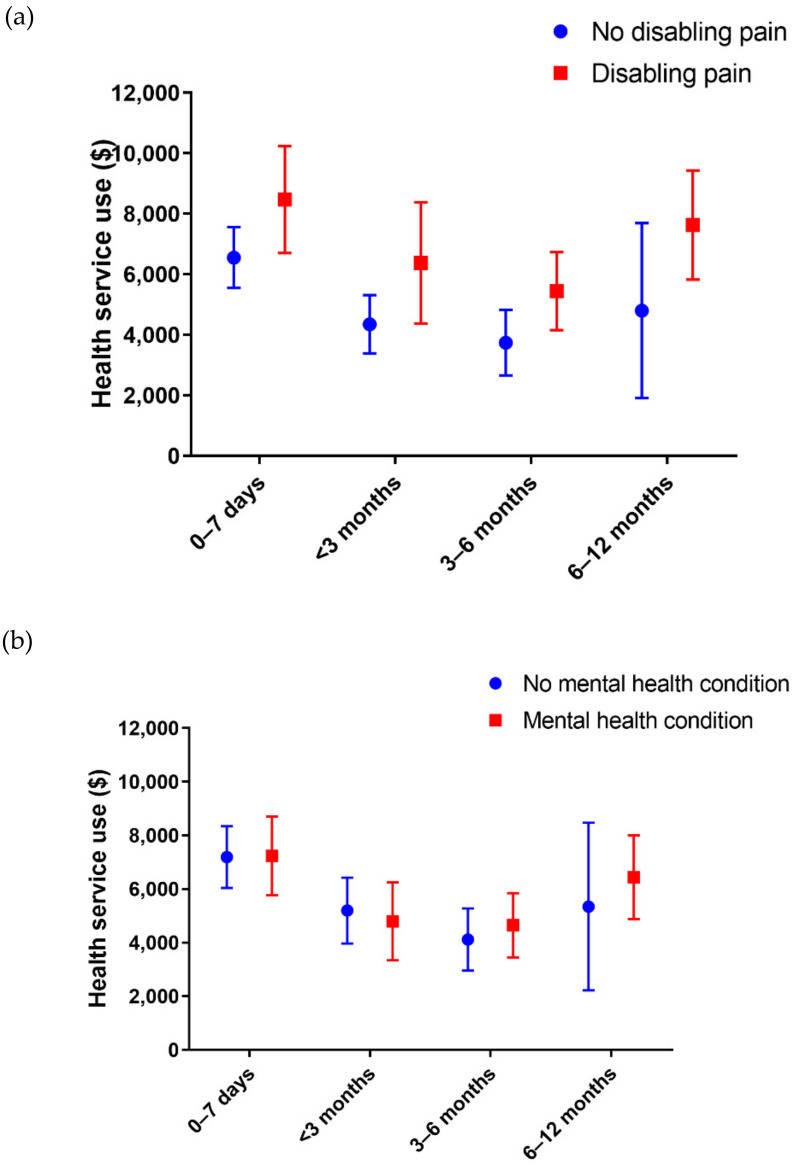
Average health service use costs ($AUD) over time for people with (**a**) disabling pain or (**b**) mental health (MH) conditions indicated relative to people without the respective condition. Mean + 95% CI.

**Figure 3:**
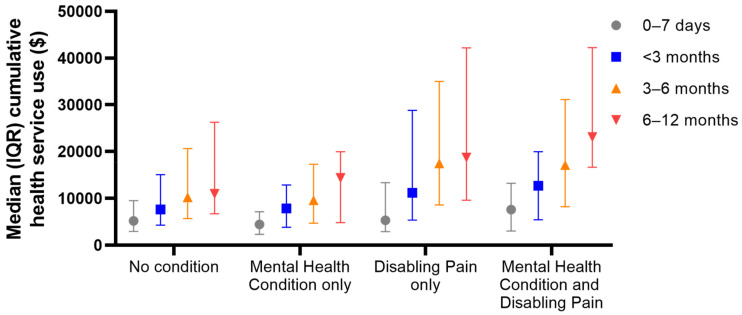
Median (IQR) cumulative health service use for people with neither condition, mental health condition only, disabling pain only or both mental health condition and disabling pain.

Higher health service use (total cost) was associated with increased odds of having disabling pain at 12 months post-injury (costs 3-6 months: OR = 1.50, 95%CI: 1.06, 2.13; costs 6–12 months: OR = 1.76, 95%CI: 1.28, 2.42). Odds of reporting symptoms of a mental health condition at 12 months post-injury were only increased for people with higher healthcare costs between 6-12 months post-injury (OR = 1.64, 95%CI: 1.23, 2.18; Figure 2 and Figure 3 and Table S2). All patients had one or more surgical medical cost in the first 12 months post-injury, and there were no significant differences in the total surgical practitioner costs between people who had chronic disabling pain (Median cost = $5687) or not (Median cost = $4622), Mann-Whitney U = 1971, *p* = 0.09, or between people who had one or more mental health condition (Median cost = $4554) or not (Median cost = $5282), Mann-Whitney U = 1618, *p* = 0.56. 

### 4.3. Multivariable Predictors of Disabling Pain

Baseline factors, including the number of body regions with moderate–severe injuries, external attribution of fault and longer hospital stay, explained 19% of the variance in the odds of having disabling pain (Cox and Snell *R^2^* = 0.14, Nagelkerke *R^2^* = 0.19; Table 4). Together with these baseline factors, use of opioid or psychotropic medications and income benefits in the first 3 months explained a total of 25% of the variance in disabling pain outcomes (Cox and Snell *R^2^* = 0.17, Nagelkerke *R^2^* = 0.24), whereas the same factors at 3–6 months explained 33% of the variance in disabling pain (Cox and Snell *R^2^* = 0.24, Nagelkerke *R^2^* = 0.33). There was no change in the variance explained when accounting for treatment between 6–12 months (Cox and Snell *R^2^* = 0.24, Nagelkerke *R^2^* = 0.33). 

Over time, the accuracy of predicting chronic pain outcomes improved, especially the sensitivity to identify people who reported chronic disabling pain, from baseline factors resulting in 70.3% accuracy (32.7% sensitivity, 89.3% specificity; AUC = 0.71, 95%CI: 0.62, 0.78) to 71.6% accuracy when accounting for claims factors in the first 3 months post-injury (40.4% sensitivity, 87.4% specificity; AUC = 0.73, 95%CI: 0.65, 0.82), and 76.8% accuracy when accounting for claims factors at 3–6 months post-injury (50.0% sensitivity, 90.3% specificity; AUC = 0.78, 95%CI: 0.70, 0.86). There was a decrease in accuracy at 6–12 months due to a reduction in specificity (total accuracy = 72.5; 51.9% sensitivity, 82.5% specificity; AUC = 0.79, 95%CI: 0.71, 0.86). The AUC figures are available in Figure S1. Characteristics at 3–6 months post-injury therefore had the greatest sensitivity and specificity to predict chronic disabling pain at 12 months after injury, which did not improve substantially when accounting for claims characteristics at 6–12 months post-injury.

### 4.4. Multivariable Predictors of Mental Health Conditions

Baseline factors, including age, sex, fault and whether impairment was sustained from the injury resulting in an impairment lump sum payment, explained 12% of the variance in the odds of having clinically elevated symptoms of at least one mental health condition (Cox and Snell *R^2^* = 0.087, Nagelkerke *R^2^* = 0.12; AUC = 0.66, 95%CI: 0.56, 0.75; Table 5 and Figure S2). While having the injury classified as causing permanent impairment within the first 12 months of injury was associated with mental health outcomes, age, sex and fault attribution were not uniquely associated with mental health outcomes. 

In addition to the baseline factors, opioid use, psychotropic medications and income benefits in the first 3 months explained a total of 16% of the variance in mental health outcomes (Cox and Snell *R^2^* = 0.12, Nagelkerke *R^2^* = 0.16; AUC = 0.68 (95%CI: 0.59, 0.77). Psychotropic medications in the first 3 months were not uniquely associated with reporting a mental health condition at 12 months. The same factors at 3–6 months post-injury explained 23% of the variance in mental health outcomes (Cox and Snell *R^2^* = 0.17, Nagelkerke *R^2^* = 0.23; AUC = 0.74 (95%CI: 0.66, 0.82), and at 6–12 months explained 28% variance in mental health outcomes (Cox and Snell *R^2^* = 0.21, Nagelkerke *R^2^* = 0.28; AUC = 0.76 (95%CI: 0.68, 0.85). The accuracy of predicting mental health outcomes improved when accounting for income payments and medication use in each time period, particularly increasing the sensitivity to correctly identify people who did develop mental health conditions from baseline adjustments. This resulted in an increase from 70.1% accuracy (42.4% sensitivity, 87.4% specificity) to 70.8% accuracy when accounting for medications and income benefits in the first 3 months (40.7% sensitivity, 89.5% specificity), 70.8% accuracy at 3–6 months (45.8% sensitivity, 86.3% specificity) and 72.7% accuracy at 6–12 months post-injury (50.8% sensitivity, 86.3% specificity). 

## 5. Discussion

This study found that one year after hospitalisation for traumatic injury, one third of people had disabling pain and more than a third of people had clinically elevated symptoms of at least one mental health condition. Less than 12 percent of people who developed a mental health condition had a prior mental health condition, and having a prior mental health condition did not increase the odds of reporting clinically significant symptoms of anxiety, depression or PTSD symptoms 12 months post-injury. More than one in five people had both disabling pain and a mental health condition. Altogether, injury, compensation and health care factors predicted pain and mental health outcomes with 70% accuracy. The accuracy for identifying disabling pain was best at 3–6 months post-injury, and there were no marked improvement in accuracy to identify mental health outcomes at 6–12 months post-injury. Both disabling pain and mental health conditions were more prevalent among people whose injury led to permanent impairment, who underwent at least one IME in the first 12 months, consulted a lawyer or were work-disabled beyond the first 6 months post-injury. These associations are most likely bidirectional given that people experiencing disabling pain or mental health conditions are probably more likely to undergo an IME, to be identified as having permanent impairment or to consult a lawyer to assist with their claim. While injury severity was positively associated with reporting disabling pain at 12 months post-injury, it was not associated with having a probable mental health condition. People who were taking psychotropic medications after the first three months post-injury, taking opioids and had work disability at 6–12 months had higher odds of having disabling pain at 12 months post-injury when adjusting for baseline characteristics. Similarly, people who were taking opioids at 3-6 months, had work disability and were taking psychotropic medications at 6–12 months post-injury had increased odds of having a mental health condition at 12 months post-injury. The present findings extend previous observations that early prescription of opioids and psychotropic medications is predictive of overall claim expense [58]. The present results suggest that medication use beyond the first three months post-injury is sensitive to aid in the identification of people who are more likely to develop disabling pain and poor mental health.

### 5.1. Injury Compensation, Pain and Mental Health

The present findings should be considered in light of the unique characteristics of the Victorian compensation schemes, particularly the transport injury compensation scheme given the majority of participants had road traffic injuries. Under the TAC compensation scheme, injured people are entitled to healthcare required to support their recovery, and to income replacement for the first 18 months post-injury if their work capacity is impaired, regardless of who was at fault for the accident. These entitlements are a stark contrast with the limited or adversarial and stressful procedures involved in seeking compensation from other schemes, particularly insurers operating within fault-based schemes which lead to much worse health, pain, mental health and work outcomes [26]. With the TAC, people who sustain permanent impairment and were not at fault are entitled to a lump sum compensation payment through common law proceedings. Moreover, people who sustain permanent impairment may be entitled to ongoing payments for loss of earning capacity after the first 18 months post-injury. Previous studies have found that TAC claimants, as with people with claims in other no-fault schemes [59], judge the scheme to be fairer and have superior health outcomes compared with fault-based compensation schemes [60]. 

While the majority of people in the present study were happy with their compensation claim, we found that the development of disabling pain or mental health conditions 12 months post-injury was associated with finding the claims process to be stressful or to cause anxiety. Moreover, people who felt that they had to keep proving the severity of their injury or disability had higher odds of disabling pain or mental health conditions. It is possible that the impacts of the injury led to the retrospective evaluation that compensation-related procedures are stressful. However, we suggest that specific compensation procedures may have exacerbated the experience of stress from compensation scheme procedures. In particular, people who had an IME had 3–4 times the odds of having disabling pain or a mental health condition at 12 months than people who did not have an IME within that time frame. We recognise that people who underwent an IME probably had more severe injuries and/or had developed disability due to the injury, persistent pain or mental health conditions, and the IME process did not necessarily cause those outcomes. However, previous research has shown that the IME process is a significant problem for clients with compensable injury, especially for people with psychological conditions [31]. This is partly due to the fact that throughout an IME clients are examined by healthcare practitioners (medical, dental or allied health) who are not familiar to them and required to repeat their medical history. Moreover, the practitioner undertaking the IME holds a significant level of power in determining the claimant’s compensation benefits. IMEs have been reported to have been used by compensation agents to justify the cessation of benefits or to expedite claim closure, which is extremely stressful to the injured person [61]. For people who already experience disabling pain and/or symptoms of posttraumatic stress, depression or anxiety, undergoing an IME may even contribute to the maintenance of worse health outcomes through exacerbation of stress mechanisms [62]. 

### 5.2. Implications for Health care and Compensation Schemes

The present findings give rise to several recommendations to improve health outcomes following compensable injury, especially processes to improve compensation experience, client screening and early intervention. First, compensation schemes could address sources of procedural stress by providing timely, clear and sufficient information about support and services that clients are entitled to. It is also important to support clients through examination-related requirements. Moreover, claim managers’ capacity to support client recovery could be enhanced. For instance, by enhancing their knowledge of how claim-related procedures can impact client stress and potentially exacerbate pain, psychopathology and disability outcomes, and receiving training in communication skills to attenuate the level of distress or scrutiny claimants feel. Further, schemes may enhance client outcomes by implementing procedures to enable real-time monitoring of claimant symptoms and treatment needs; altering procedures that require the engagement of a lawyer; and improving the efficiency with which claims are handled and services are provided [30,63,64,65]. Segmentation of claims so that clients with specific needs or complexity profiles are managed by specialist teams or case managers would also be beneficial in enhancing timely support and reducing redundancy. Finally, various strategies may reduce the stressfulness of IMEs, especially for people who have pain or mental health symptoms [31]. For instance, reassuring clients that the examinations are not intended to delegitimise or trivialise their injury or disability but that they are a necessary process to ensure that they receive the right level and type of ongoing support to get their life back on track. Moreover, sharing of medical information with and between examiners may minimise the degree to which clients have to repeat their clinical history. 

### 5.3. Client Screening and Treatment

Ideally, clients at risk of developing chronic pain should be identified before pain becomes chronic and disabling. Characteristics that we evaluated as potential screening criteria included the initial injury characteristics (i.e., hospitalisation, injury severity, impairment), continued use of prescription opioid analgesics, psychotropic agents and work disability. Given that nearly all of these characteristics are available in compensation scheme records, an automated process could be implemented to identify clients at risk of poor recovery, triggering a claim and healthcare review by a case manager; referral for medication review by a clinical panel, pain physician or psychiatrist; or priority early access to multidisciplinary interventions to prevent those problems from becoming persistent. However, vigilance to identify clients at risk should be maintained beyond the early post-injury period as new sources of stress are likely to arise over time, or from the compensation claim, as the impacts of injury on work, family and social roles are realised [66].

In our multivariate prediction models, while specificity was high, accuracy in predicting chronic pain, alongside baseline factors, was greatest at 3–6 months post-injury. Considering the present cohort sustained injuries that were serious enough to require hospitalisation and surgical procedures for serious injuries, it is likely that specific factors (e.g., taking opioids, health care use) were not uniquely predictive of pain/mental health outcomes during the early period while the injury was still stabilising and health care needs remained relatively high. Thus, after injury resulting in a period of hospitalisation, we recommend that screening should take into account not only baseline characteristics but also healthcare use before 3 months in order to identify which clients are likely to develop disabling pain or mental health conditions. Clients with minor injuries may benefit from different screening periods and criteria. Moreover, the “ideal” time to screen for risk of persistent problems may include different criteria in early timeframes that were not available in the present study.

Once clients at risk are identified, proactive early access to treatment should be provided in order to change the recovery course before outcomes become persistent [67,68]. Ideally, this should begin within the first weeks and month post-injury, and the present study shows that if people continue to have ongoing problems with pain or mental health, as indicated by ongoing treatment access beyond the first 3–6 months post-injury, they are at increased risk of having clinically significant or disabling pain or mental health problems at 12 months post-injury. There is some evidence to suggest that early multidisciplinary interventions for pain can reduce the development of chronic disabling pain and associated health care costs [69,70], facilitate return to work [67,71] and improve general mood, health and quality of life [72]. Early education about stress responses, exposure and challenging of unrealistic thoughts for people with heightened acute stress has also been shown to prevent transition to PTSD [73,74]. Moreover, proactive collaborative care interventions have been found to reduce the incidence and severity of mental outcomes following injury [75]. By contrast, early information-based interventions may exacerbate symptoms of PTSD post-injury [76], and the therapeutic impacts of some interventions, such as debriefing, have been found to be detrimental [77]. 

Considering the close association between persistent pain and mental health conditions, a therapeutic approach that targets common symptoms that underlie maladaptive cognitions and behaviours should be considered as part of routine practice after injury, with additional treatment for specific conditions, such as prolonged exposure for people diagnosed with PTSD. Integrated multidisciplinary care to enable medication review and to develop active (e.g., participating in therapies, working toward resuming activities) rather than passive (e.g., withdrawing from activities and relying on receiving medication to manage symptoms) coping strategies have also been recommended [78].

### 5.4. Limitations

Some limitations to the present results should be considered. First, all participants had moderate to severe injuries resulting in hospitalisation, and the findings may not generalise to people with minor soft tissue injures or gradual onset musculoskeletal conditions that are more common in work compensation claims. Moreover, both compensation schemes had no-fault entitlements and may not generalise to fault-based schemes where claim-related stress and dissatisfaction are more complex and pronounced [79]. That said, the characteristics of the cohort were similar to other compensation cohort studies, in which approximately three quarters are male, with relatively low education levels and but high participation in work pre-injury [18]. Although sex is known to influence perceptions of pain after injury [6,13], we did not include this as a covariate due to the study’s small sample size, the predominance of males (75.2%) and the fact that the univariate analyses showed that sex did not increase the odds of reporting disabling pain. Some characteristics known to be associated with the development of persistent pain such as baseline pain severity after injury or surgery [1,80] were not available, and prescription medications during the hospitalisation and early post-discharge period are often not reliably captured in the compensation claims and could not be used as a proxy marker of baseline pain. The follow-up was restricted to the first 12 months post-injury, which limited the complexity of the multivariate analyses and potential to examine longer term outcomes. The small sample size also limited our capacity to test the specificity and sensitivity across multiple subsets within the sample. Future large-scale, multisite longitudinal studies should therefore be undertaken to enable the inclusion of all important predictors of pain and mental health outcomes and to enable validation of the prediction models. The results may be specific to this cohort and should be replicated in larger samples and over an extended timeframe. 

The study included robust routinely collected data on treatments received through the compensation claim, which could be used by the respective compensation schemes to identify clients at risk of pain or mental health conditions. However, we assumed that people who filled a prescription then took the respective medication. The claims data may not have included all prescription medications for pain and mental health given that in Australia most prescribed medications are subsidised under the Pharmaceutical Benefits Scheme, making them affordable and relatively cheap; therefore, not all claimants seek reimbursement for every medication from their compensation claim [35,36]. Likewise, we could not account for additional health services accessed that were not covered by the compensation claim. Finally, as most participants were employed at the time of injury they most likely initially used their sick leave and annual leave entitlements before claiming income benefits. 

## 6. Conclusions

Patients with disabling pain and mental health conditions at 12 months post-injury could be identified with a high level of accuracy from their baseline demographic and injury characteristics, and compensation-related characteristics within the first 3–6 months following injury, especially the use of opioid and/or psychotropic medications. In particular, the use of opioid or psychotropic medications had excellent capacity to predict pain and mental health outcomes by 3–6 months post-injury, highlighting a valuable opportunity to augment recovery through timely and targeted interventions for people still using medications at 3-months post-injury. Negative compensation-related procedural experiences, IMEs and lawyer involvement in the claim were all associated with poorer outcomes. Wlthough we recognise that these associations are probably bidirectional, they should be considered proxy indicators of clients at risk. We suggest several strategies to improve injury outcomes and to identify people at risk of poor recovery early post-injury, and implementing time-sensitive, multidisciplinary pain, medication and psychosocial interventions for people at risk of disabling pain or mental health conditions.

## Figures and Tables

**Table 1:** Summary of Medical, Paramedical and Pharmaceutical Items (Total) Received by the Sample in the First 12 Months after Injury.

	Number of Items	Total Cost ($AUD) ^a^
Medical ^b^		
Psychiatry	73	$14,630
Surgery-related doctor fees	1146 ^c^	$889,840 ^d^
Pathology tests	5905	$183,393
Imaging	3290	$551,096
General Practitioner	1671	$116,451
Specialist consultations	2462	$291,385
Paramedical		
Rehabilitation and return to work programs	404 ^e^	$182,311
Physical therapies ^f^	8737	$443,440
Psychology	825 ^e^	$87,034
Occupational therapy	2285	$136,059
Other allied health services ^g^	1762	$127,876
Aids, equipment, home/vehicle modifications ^h^	1711^h^	$462,116
Pharmaceutical items ^i^		
… for mental health (psychotropic medications)	216	$3968
… opioids	691	$14,554
… non-opioid analgesics	458	$991

^a^ Inflated to June 2014 value paid by the TAC; ^b^ medical services were classified using the Medicare Benefits Schedule (MBS) service codes; ^c^ note that each surgery may include multiple items per treatment episode; ^d^ these costs only include the doctor fees charged for MBS surgery items and do not include additional cost of care related to the surgery (e.g., accommodation, theatre, surgery items); ^e^ patients could receive multiple items per episode; ^f^ physical therapies includes physiotherapy, exercise physiology, physical education, chiropractic and osteopathy; ^g^ other allied health includes consultations and services from speech therapy, social work, podiatry, dental, optical, acupuncture and nursing; ^h^ note that these often involve a concurrent occupational therapy assessment; ^i^ pharmaceutical items do not include over-the-counter medications and were classified according to the Anatomical Therapeutic Chemical (ATC) Classification System [43] for mental health medications (N05B, N05C, N05CH, N06A, N06B, N07B, NO5A), opioids (N02A) and other analgesics (M01A, M03B, N02B, N03A), including low dose amitriptyline and duloxetine [N06A], which are more often prescribed for neuropathic pain rather than as an antidepressant.

**Table 2:** Participant Demographic and Injury Characteristics and Prevalence of Disabling Pain or Probable Mental Health Condition at 12 Months Post-Injury.

		Total	Disabling Pain	Mental Health Condition
Characteristic		*N* (%)	*N* (%)	*N* (%)
Sex	Male	118 (75.2)	36 (67.9)	41 (68.3)
	Female	39 (24.8)	17 (32.1)	19 (31.7)
Age	18–24 Years	28 (17.8)	10 (18.9)	16 (26.7)
	25–34 Years	16 (10.2)	<5	<5
	35–44 Years	39 (24.8)	13 (24.5)	15 (25.0)
	45–54 Years	30 (19.1)	13 (24.5)	9 (15.0)
	55+ Years	44 (28.0)	14 (26.4)	16 (26.7)
Education	University	35 (22.3)	11 (20.8)	9 (15.0)
	Diploma	62 (39.5)	15 (28.3)	21 (35.0)
	Year 12	27 (17.2)	10 (18.9)	16 (26.7)
	< Year 12	33 (21.0)	17 (32.1)	14 (23.3)
Work before Injury	Employed	133 (84.7)	41 (77.4)	45 (75.0)
	Unemployed	24 (15.3)	12 (22.6)	15 (25.0)
Work Status, 12 Months	Returned to Work	105 (72.4)	22 (46.8)	34 (61.8)
	Not Returned to Work	40 (27.5)	25 (53.2)	21 (38.2)
Remoteness	Major Cities	106 (67.5)	36 (67.9)	42 (70.0)
	Regional	51 (32.5)	17 (32.1)	18 (30.0)
Comorbid Conditions at 12 Months, Self-Report	None	102 (65.0)	30 (56.6)	41 (68.3)
	≥1 Comorbidity	55 (35.0)	23 (43.4)	19 (31.7)
Prior Mental Health Condition	No	141 (89.8)	49 (92.5)	53 (88.3)
	Yes	16 (10.2)	<5	7 (11.7)
Prior Substance Use Condition	No	148 (94.3)	51 (96.2)	54 (90.0)
	Yes	9 (5.7)	<5	6 (10.0)
Engaged a Lawyer within 12 Months Post-Injury	No	99 (63.1)	21 (40.4)	29 (49.2)
	Yes	55 (35.0)	31 (59.6)	30 (50.8)
Compensation Scheme *	TAC	134 (85.4)	44 (84.6)	49 (83.1)
	WSV *	23 (14.6)	9 (17.3)	11 (18.6)
Self at Fault	No	103 (66.5)	39 (75.0)	44 (74.6)
	Yes	52 (33.5)	13 (25.0)	15 (25.4)
Impairment Payment Received	No	113 (72.0)	25 (47.2)	35 (58.3)
	Yes	44 (28.0)	28 (52.8)	25 (41.7)
AIS, > = 1 Moderate–Severe Injury	1. Head/Face	58 (36.9)	21 (39.6)	23 (38.3)
	2. Face	40 (25.5)	17 (32.1)	20 (33.3)
	3. Neck	8 (5.1)	7 (13.2)	<5
	4. Thorax	89 (56.7)	34 (64.2)	32 (53.3)
	5. Abdomen	39 (24.8)	16 (30.2)	18 (30.0)
	6. Spine	64 (40.8)	27 (50.9)	26 (43.3)
	7. Upper Extremity	72 (45.9)	25 (47.2)	27 (45.5)
	8. Lower Extremity	95 (60.5)	33 (62.3)	35 (58.3)
	9. Unspecified	14 (8.9)	7 (13.2)	6 (10.0)
Discharge Location	Home	83 (52.9)	21 (39.6)	28 (46.7)
	Rehabilitation	74 (47.1)	32 (60.4)	32 (53.3)

* One of the WSV claimants also had a TAC claim for the same injury as they sustained a serious injury and some of their care was supported via the TAC independence claims branch, who support claimants with catastrophic injuries (e.g., paraplegia, quadriplegia or serious brain injury). For the purpose of analyses, this person was classified as a WSV claimant as the injury occurred while working. Abbreviations: AIS = abbreviated injury severity; TAC = Transport Accident Commission; WSV = WorkSafe Victoria.

**Table 3:** Prevalence of Pain, Pain Disability and Mental Health Condition Criteria.

	Criteria Met
Condition Type	*N* (%)
**Chronic pain condition**	
Pain Severity ≥4	55 (35.0)
Pain Interference ≥4	64 (40.8)
RMDQ ≥7	87 (55.4)
CP Condition: Severe Pain AND High Pain Interference or Disability	53 (33.8)
**Mental Health Conditions**	
Anxiety (≥11)	36 (22.9)
Depression (≥11)	26 (16.6)
PTSD (≥36)	69 (43.9)
PTSD (DSM-5, Criteria A, B, C, D & E)	50 (31.8)
PTSD (≥36 AND all Cluster Criteria)	50 (32.1)
Anxiety or Depression or PTSD Dual Criteria	60 (38.5)
**Chronic Pain and Mental Health Condition**	36 (23.1)

Abbreviations: DSM = Diagnostic and Statistical Manual; PTSD = posttraumatic stress disorder; RMDQ = Roland–Morris Disability Questionnaire.

**Table 4:** Binary Logistic Multivariable Regression Coefficients of Early Prognostic Factors for Chronic and Disabling Pain 12 Months after Compensable Injury.

		Total	Disabling Pain	Odds of Reporting Disabling Pain	
Predictor		*N* (%)	*N* (%)	OR (95% CI)	AOR (95% CI)
AIS Region Count		--	--	1.03 (1.00, 1.07)	1.22 (1.21, 1.23)
Fault	Self at Fault	52 (33.1)	13 (25.0)	1.00	1.00
	Not at Fault	103 (65.6)	39 (37.9)	1.83 (0.87, 3.84)	1.88(1.83; 1.93)
Hospital Length of Stay	12 days	29 (18.5)	6 (20.7)	1.00	1.00
	3–6 days	54 (34.4)	21 (38.9)	2.44 (0.85, 6.99)	2.10 (2.03, 2.18)
	7–13 days	42 (26.8)	8 (19.0)	0.90 (0.28, 2.95)	0.62 (0.59, 0.64)
	≥14 days	32 (20.4)	18 (56.3)	4.93 (1.58, 15.38)	3.39 (3.25, 3.53)
<3 Months Post-Injury ^†^	Income Benefits	114 (72.6)	40 (35.1)	1.25 (0.59, 2.66)	1.22 (0.52, 2.83)
	Opioids	58 (36.9)	24 (41.4)	1.70 (0.86, 3.36)	1.44 (0.62, 3.35)
	Psychotropic Medications	15 (9.6)	10 (66.7)	4.61 (1.49, 14.28)	2.89 (0.72, 11.54)
3–6 Months Post-Injury ^†^	Income Benefits	95 (60.5)	38 (40.0)	2.09 (1.03, 4.26)	1.71 (0.73, 4.02)
	Opioids	36 (22.9)	20 (55.6)	3.33 (1.54, 7.20)	1.19 (0.42, 3.39)
	Psychotropic medications	17 (10.9)	14 (82.4)	12.09 (3.29, 44.37)	9.08 (1.89, 43.64)
6–12 Months Post-Injury ^†^	Income Benefits	69 (43.9)	36 (51.4)	4.36 (2.15, 8.85)	2.85 (1.22, 6.62)
	Opioids	28 (17.8)	20 (71.4)	7.27 (2.93, 18.07)	3.84 (1.15, 12.84)
	Psychotropic medications	17 (10.9)	12 (70.6)	5.79 (1.92, 17.50)	1.32 (2.93, 5.91)

^†^ Controlling for baseline factors. In the <3-month timeframe, data from the first seven days post-injury were not included as medication data are typically incomplete due to hospitalisation, and income replacement may not be covered if participants have first used sick leave entitlements and/or are not yet entitled to compensable income replacement, which only commences after the first five days post-injury. Abbreviations: AIS = Abbreviated Injury Severity, AOR = Adjusted Odds Ratio, CI = Confidence Interval, N = Number, OR = Odds Ratio.

**Table 5:** Binary Logistic Multivariable Regression Coefficients of Early Prognostic Factors for Symptoms of a Mental Health Condition after Compensable Injury.

		Total	MH Condition	Odds of Reporting Symptoms of a MH Condition	
Predictor		*N* (%)	*N* (%)	OR (95% CI)	AOR (95% CI)
Age at Injury	(Years)	--	--	0.98 (0.96, 1.00)	0.98 (0.96, 1.01)
Sex	Male	118 (75.2)	41 (35.0)	1.00	1.00
	Female	39 (24.8)	19 (48.7)	1.76 (0.86, 3.67)	1.47 (0.68, 3.20)
Fault	Self at Fault	52 (33.1)	15 (29.4)	1.00	1.00
	Not at Fault	103 (65.6)	44 (42.7)	1.79 (0.87, 3.67)	1.82 (0.86, 3.86)
Impairment	No	113 (72.0)	35 (31.3)	1.00	1.00
	Yes	44 (28.0)	25 (56.8)	2.89 (1.41, 5.94)	2.89 (1.37, 6.09)
<3 Months Post-Injury ^†^	Income Benefits	114 (72.6)	45 (39.5)	1.17 (0.56, 2.45)	1.08 (0.48, 2.41)
	Opioids	58 (36.9)	27 (46.6)	1.72 (0.88, 3.34)	1.13 (0.51, 2.53)
	Psychotropic Medications	15 (9.6)	11 (73.3)	5.16 (1.56, 17.07)	3.62 (0.92, 14.27)
3–6 Months Post-Injury ^†^	Income Benefits	95 (60.5)	43 (45.3)	2.14 (1.07, 4.27)	1.49 (0.68, 3.27)
	Opioids	36 (22.9)	24 (66.7)	4.67 (2.11, 10.34)	2.93 (1.10, 7.83)
	Psychotropic medications	17 (10.9)	13 (76.5)	6.36 (1.97, 20.59)	2.32 (0.57, 9.38)
6–12 Months Post-Injury ^†^	Income Benefits	69 (43.9)	40 (57.1)	4.40 (2.21, 8.76)	3.24 (1.48, 7.10)
	Opioids	28 (17.8)	17 (60.7)	3.06 (1.32, 7.09)	0.49 (0.14, 1.73)
	Psychotropic medications	17 (10.9)	14 (82.4)	9.44 (2.58, 34.48)	9.58 (1.92, 47.69)

^†^ Controlling for baseline factors. In the < 3-month timeframe, data from the first seven days post-injury were not included as medication data are typically incomplete due to hospitalisation, and income replacement may not be covered if participants have first used up their sick leave entitlements and/or are not yet entitled to compensable income replacement. Abbreviations: AOR = Adjusted Odds Ratio, CI = Confidence Interval, MH = Mental Health, N = Number, OR = Odds Ratio.

## Supplementary Materials

The following are available online at https://www.mdpi.com/1660-4601/17/19/7320/s1, **Table S1**: Source of each variable included in the analyses; **Table S2**: Univariate logistic regression coefficient for the association between demographics, health- and injury-related characteristics and disabling pain, mental health condition and injury severity 12 months after compensable injury; **Table S3**: Univariate logistic regression coefficient for the association between compensation scheme experience and disabling pain, mental health condition and injury severity 12 months after compensable injury; **Figure S1**: Area under the curve for the prediction of disabling pain when taking into account (a) the baseline model and baseline characteristics in addition to claims characteristics in the first three months post-injury (b); 3–6 months post-injury (c); and 6–12 months post-injury (d); **Figure S2**: Area under the curve for the prediction of mental health conditions when taking into account (a) the baseline model and baseline characteristics in addition to claims characteristics in the first three months post-injury (b); 3–6 months post-injury (c); and 6–12 months post-injury (d).
