# Supplementary material for: Prognostic Role of Demographic, Injury and Claim Factors in Disabling Pain and Mental Health Conditions 12 Months after Compensable Injury
Source: Int J Environ Res Public Health. 2020 Oct 7;17(19):7320. doi: 10.3390/ijerph17197320 (PMC7579145; doi:10.3390/ijerph17197320)
Supplement: Supplementary file 1 [file ijerph-17-07320-s001.pdf]

Table S1. Source of each variable included in the analyses.

	Data source		
	VSTR or VOTOR	Study Questionnaires	Compensation Research Database
Demographics			
Sex	Yes		
Age	Yes		
Education	Yes		
Work before injury	Yes		
Work status, 12-months	Yes	Yes	
Remoteness of residential location	Yes		
Health			
Comorbid conditions at 12-months		Yes	
Prior mental health condition	Yes		
Prior substance use condition	Yes		
Injury-related characteristics			
AIS, > = 1 moderate-severe injury	Yes		
Hospital length of stay	Yes		
Discharge location	Yes		
Compensation-related			
Compensation scheme	Yes		
Engaged a lawyer within 12-months post-injury		Yes	
Self at Fault		Yes	
Compensation experience		Yes	
Impairment payment received			Yes
Independent Medical Examination			Yes
Total healthcare costs			Yes
Medical, paramedical and pharmaceutical items			Yes
Income benefits			Yes
Opioid medication use			Yes
Psychotropic medication use			Yes
Outcomes, pain			
Pain severity (Brief Pain Inventory)		Yes	
Pain interference (Brief Pain Inventory)		Yes	
Pain-related disability (Roland Morris Disability Questionnaire)		Yes	
Outcomes, Mental health			
Anxiety (Hospital Anxiety and Depression Scale)		Yes	
Depression (Hospital Anxiety and Depression Scale)		Yes	
PTSD (Posttraumatic Checklist)		Yes	

Table S2. Univariate logistic regression coefficients for the association between demographics, health and injury-related characteristics and disabling pain, mental health condition and injury severity 12-months after compensable injury.

			**Disabling pain**	**Mental health condition**
Predictor		N (%)[+]	OR (95% CI)	OR (95% CI)
Pre-injury demographics and health				
Age	(continuous)		1.00 (0.98, 1.03)	0.98 (0.96, 1.00)
Sex	Male<br>Female	118 (75.2)<br>39 (24.8)	1.00<br>1.76 (0.84, 3.71)	1.00<br>1.76 (0.85, 3.67)
Education level	University degree<br>Did not complete secondary school<br>Secondary school<br>Diploma	35 (22.3)<br>33 (21.0)<br>27 (17.2)<br>62 (39.5)	1.00<br>2.32 (0.83, 6.22)<br>1.28 (0.45, 3.70)<br>0.70 (0.28, 1.75)	1.00<br>2.05 (0.73, 5.72)<br>4.04 (1.37, 11.92)<br>1.42 (0.53, 3.59)
Employed prior to injury	Yes<br>No	133 (84.7)<br>24 (15.3)	1.00<br>2.09 (0.64, 6.81)	1.00<br>1.16 (0.35, 3.82)
Remoteness	Major cities<br>Regional/Remote	106 (67.5)<br>51 (32.5)	1.00<br>0.97 (0.48, 1.97)	1.00<br>0.86 (0.43, 1.72)
Comorbidities at 12-months	None<br>One or more	102 (65.0)<br>55 (35.0)	1.00<br>1.73 (0.87, 3.42)	1.00<br>0.77 (0.39, 1.53)
Neighbourhood socioeconomic status, IRSD decile	(continuous)	157	0.27 (0.82, 1.06)	0.97 (0.86, 1.09)
Injury severity				

			Disabling pain	Mental health condition
Predictor		N (%)+	OR (95% CI)	OR (95% CI)
AIS sum	(continuous)	157	1.03 (1.00, 1.07)	0.99 (0.96, 1.02)
Number of body regions with one or more mod-severe injury	(continuous)	157	1.37 (1.06, 1.76)	1.01 (0.79, 1.28)
Length of hospital stay	1-2 days	29 (18.5)	1.00	1.00
	3-6 days	54 (34.4)	2.44 (0.85, 6.99)	0.79 (0.32, 1.99)
	7-13 days	42 (26.8)	0.90 (0.28, 2.95)	0.64 (0.24, 1.70)
	≥14 days	32 (20.4)	4.93 (1.58, 15.38)	0.52 (0.18, 1.50)
Discharge destination	Home	83 (52.9)	1.00	1.00
	Rehabilitation	74 (47.1)	2.25 (1.15, 4.42)	1.47 (0.77, 2.81)
Outcomes and Compensation-related characteristics				
Work status, 12-months	Returned to work	105 (72.4)	1.00	1.00
	Not returned to work	40 (27.5)	6.29 (2.89, 13.34)	2.94 (1.43, 6.06)
Impairment payment within 12-months	No	113 (72.0)	1.00	1.00
	Yes	44 (28.0)	6.16 (2.89, 13.15)	2.89 (1.41, 5.94)
Self at fault	Yes	52 (33.5)	1.00	1.00
	No	103 (66.5)	1.83 (0.87, 3.84)	1.79 (0.87, 3.67)
IME in first 12 months post injury	No	124 (79.0)	1.00	1.00
	Yes	33 (21.0)	3.05 (1.39, 6.72)	4.47 (1.97, 10.14)

			Disabling pain	Mental health condition
Predictor		N (%)[+]	OR (95% CI)	OR (95% CI)
Engaged a lawyer within 12-months post-injury	No	99 (63.1)	1.00	1.00
	Yes	55 (35.0)	4.80 (2.34, 9.84)	2.86 (1.44, 5.67)
Total health service use costs*				
<3 months	(continuous, log transformed)	157	1.48 (0.87, 2.51)	1.00 (0.63, 1.62)
3-6 months	(continuous, log transformed)	157	1.50 (1.06, 2.13)	1.17 (0.88, 1.57)
6-12 months	(continuous, log transformed)	157	1.76 (1.28, 2.42)	1.64 (1.23, 2.18)

[+] Analyses examining mental health outcomes were missing one participant who was included in the pain analyses. * Note that the total health service use analysis included a log transformed continuous variable. Therefore the ORs show that with every unit increase in healthcare cost there is a corresponding relative increase in odds of the respective outcome (e.g., every unit increase in healthcare costs at 3-6 months is associated with a 50% increased odds of having disabling pain at 12-months).

Table S3. Univariate logistic regression coefficient for the association between compensation scheme experience and disabling pain, mental health condition and injury severity 12-months after compensable injury.

			Disabling pain	Mental health condition
Predictor		N (%)+	OR (95% CI)	OR (95% CI)
Compensation system experience in first 12 months post-injury*	Negative compensation procedural experience	157	1.78 (1.29, 2.45)	2.08 (1.49, 2.91)
	Compensation supported recovery	157	0.53 (0.35, 0.80)	0.45 (0.30, 0.70)
	Positive compensation procedural experience	157	0.70 (0.47, 1.05)	0.43 (0.28, 0.67)
I feel like I have to keep proving my injury and/or disability	Disagree/strongly disagree	72 (48.6)	1.00	1.00
	Neither agree or disagree	11 (7.4)	2.92 (0.79, 10.81)	6.53 (1.69, 25.31)
	Agree/strongly agree	65 (43.9)	3.00 (1.43, 6.28)	4.09 (1.94, 8.66)
The claims process is stressful and causes me anxiety	Disagree/strongly disagree	23 (15.3)	1.00	1.00
	Neither agree or disagree	42 (28.0)	2.25 (0.91, 5.55)	1.66 (0.67, 4.09)
	Agree/strongly agree	85 (53.8)	4.54 (2.00, 10.31)	6.33 (2.76, 14.56)
I am happy with my compensation claim	Agree/strongly agree	42 (28.0)	1.00	1.00
	Neither agree or disagree	32 (21.3)	2.28 (1.03, 4.83)	2.15 (1.00, 4.61)
	Disagree/strongly disagree	76 (50.7)	1.73 (0.66, 4.45)	2.58 (1.00, 6.61)

+ Analyses examining mental health outcomes were missing one participant who was included in the pain analyses. * The mean score on the compensation experience subscales were included in these analyses, which have a potential score range from zero to five. The OR indicates that for every one point increase in the mean score the relative odds of having disabling pain or mental health conditions is increased or decreased.

(a)

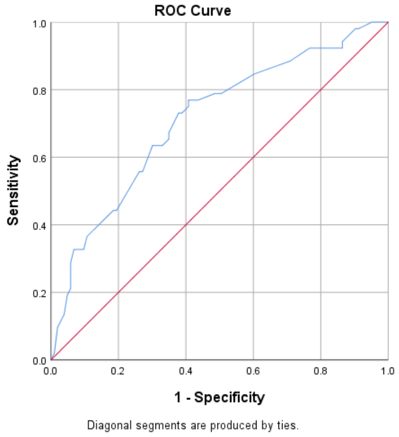

AUC=0.71 (95%CI: 0.62, 0.78)

(b)

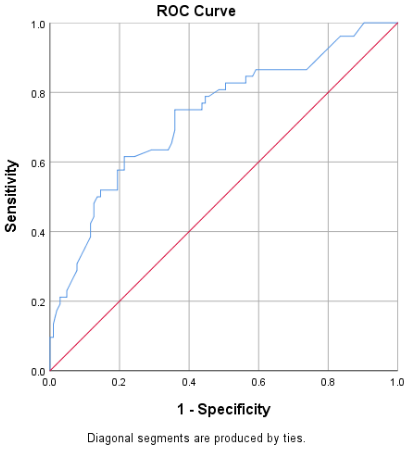

AUC=0.73 (95%CI: 0.65, 0.82)

(c)

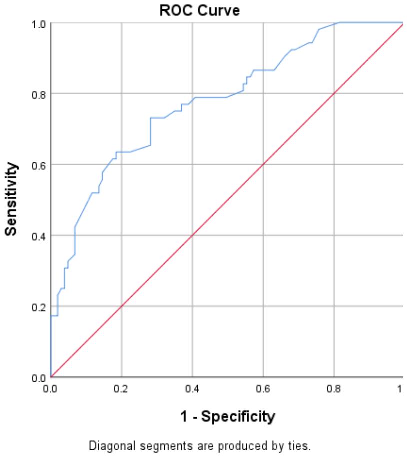

AUC=0.78 (95%CI: 0.70, 0.86)

(d)

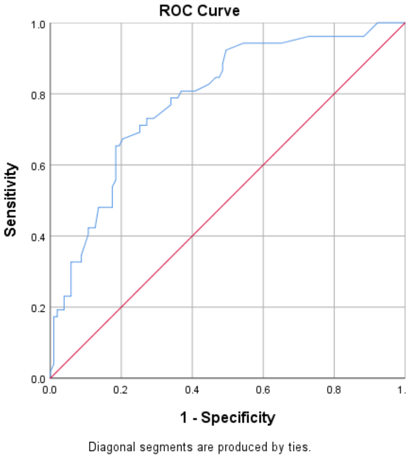

AUC=0.79 (95%CI: 0.71, 0.86)

Figure S1. Area Under the Curve for the prediction of disabling pain when taking into account (a) the baseline model, and baseline characteristics in addition to claims characteristics in the first three months post-injury (b); 3-6 months post-injury (c); and 6-12 months post-injury (d).

(a)

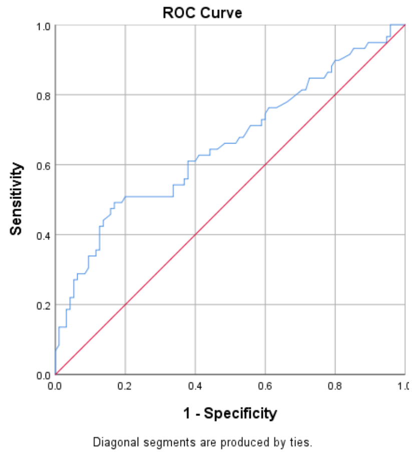

AUC=0.66 (95%CI: 0.56, 0.75)

(b)

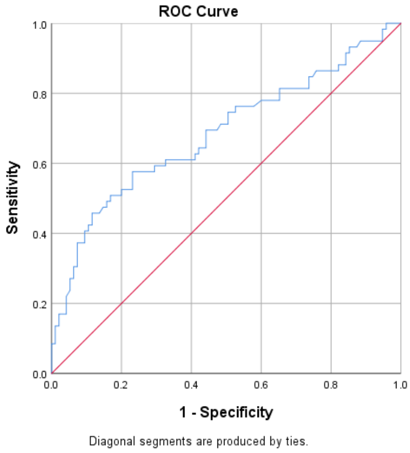

AUC=0.68 (95%CI: 0.59, 0.77)

(c)

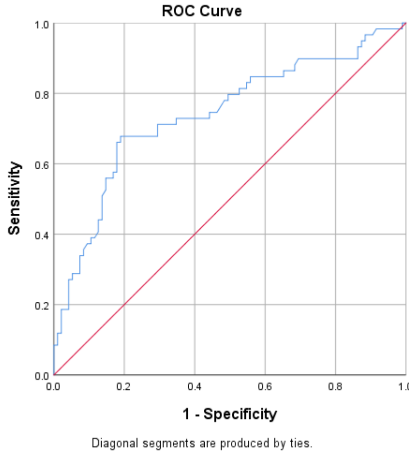

AUC=0.74 (95%CI: 0.66, 0.82)

(d) 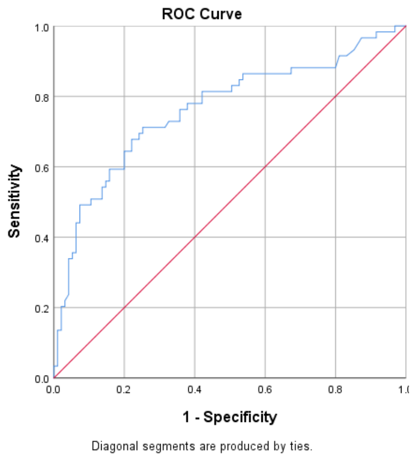 AUC=0.76 (95%CI: 0.68, 0.85)

Figure S2. Area Under the Curve for the prediction of mental health conditions when taking into account (a) the baseline model, and baseline characteristics in addition to claims characteristics in the first three months post-injury (b); 3-6 months post-injury (c); and 6-12 months post-injury (d).
